# Small Scale Multi-Object Segmentation in Mid-Infrared Image Using the Image Timing Features–Gaussian Mixture Model and Convolutional-UNet

**DOI:** 10.3390/s25113440

**Published:** 2025-05-30

**Authors:** Meng Lv, Haoting Liu, Mengmeng Wang, Dongyang Wang, Haiguang Li, Xiaofei Lu, Zhenhui Guo, Qing Li

**Affiliations:** 1 Beijing Engineering Research Center of Industrial Spectrum Imaging, School of Automation and Electrical Engineering, University of Science and Technology Beijing, Beijing 100083, China; 2 National Key Laboratory of Human Factors Engineering, China Astronaut Research and Training Center, Beijing 100094, China; 3 Jiuquan Satellite Launch Center, Jiuquan 732750, China

**Keywords:** mid-infrared image, image segmentation, Gaussian Mixture Model (GMM), UNet, grassland monitoring

## Abstract

The application of intelligent video monitoring for natural resource protection and management has become increasingly common in recent years. To enhance safety monitoring during the grazing prohibition and rest period of grassland, this paper proposes a multi-object segmentation algorithm based on mid-infrared images for all-weather surveillance. The approach integrates the Image Timing Features–Gaussian Mixture Model (ITF-GMM) and Convolutional-UNet (Con-UNet) to improve the accuracy of target detection. First, a robust background modelling, i.e., the ITF-GMM, is proposed. Unlike the basic Gaussian Mixture Model (GMM), the proposed model dynamically adjusts the learning rate according to the content difference between adjacent frames and optimizes the number of Gaussian distributions through time series histogram analysis of pixels. Second, a segmentation framework based on Con-UNet is developed to improve the feature extraction ability of UNet. In this model, the maximum pooling layer is replaced with a convolutional layer, addressing the challenge of limited training data and improving the network’s ability to preserve spatial features. Finally, an integrated computation strategy is designed to combine the outputs of ITF-GMM and Con-UNet at the pixel level, and morphological operations are performed to refine the segmentation results and suppress noises, ensuring clearer object boundaries. The experimental results show the effectiveness of proposed approach, achieving a precision of 96.92%, an accuracy of 99.87%, an intersection over union (IOU) of 94.81%, and a recall of 97.75%. Furthermore, the proposed algorithm meets real-time processing requirements, confirming its capability to enhance small-target detection in complex outdoor environments and supporting the automation of grassland monitoring and enforcement.

## 1. Introduction

**Figure 1:**
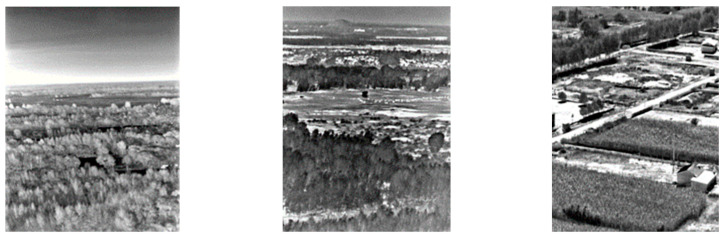
Mid-infrared image samples of grassland monitoring.

With advances in intelligent video surveillance, natural resource conservation and management face new opportunities and challenges. Effective monitoring and management of vast grassland resources have become an urgent problem [1]. Grassland resource law enforcement mainly focuses on regulating violations of grazing bans and rest period rules [2]. This paper specifically explores the challenges associated with video-based intelligent monitoring for natural resource supervision, using grassland law enforcement during grazing prohibition periods as a case study. The grassland ecosystem is highly dynamic and subject to varying weather conditions, often windy, cloudy, or foggy, which affects the imaging performance of surveillance system. Moreover, the cost problem of experimental systems in outdoor grassland areas must be considered. While visible-light cameras can produce clear images, they fail to meet the needs of all-weather operation. Near-infrared images are qualified for all-weather applications, but they cannot adapt to complex lighting variations over medium to long distances [3]. Given these limitations, this study employs mid-infrared camera, which has low dependence on ambient light sources, strong fog penetration, and all-weather imaging performance. The monitoring data samples of mid-infrared imaging system are shown in Figure 1.

At present, the traditional mobile patrol is an important regulatory method in grassland resources law enforcement monitoring. However, it demands substantial investments in manpower, material, and finance, leading to inefficiencies. Therefore, numerous scholars have focused their research on intelligent video monitoring solutions. Barbedo et al. [4] proposed a method for counting cattle based on unmanned aerial images of calves of the Nelore and Canchim breeds, and the proposed structure achieved more than 90% accuracy under different conditions and contexts. Sarwar et al. [5] used a drone to capture aerial footage from a sheep farm in Pirinoa, New Zealand and explored different Fully Convolutional Networks (FCNs) and Convolutional Neural Networks (CNNs) to detect and count sheep. The results showed that the UNet-Mean Shift (UNet-MS) model outperformed other networks. Sant’Ana et al. [6] performed hyperpixel segmentation of mixed breed sheep images of Texel and Santa Inês breeds to separate them from image background, using four architectures to train the model; the best result presented was the one using the DenseNet201 technique with an F-value of 0.928. Ayub et al. [7] collected sheep video data from a farm in Pakistan and used a pre-trained You Only Look Once version 5 (YOLOv5) model for detection and activity classification. The statistical results proved the robustness of proposed method.

Most of the previous studies rely on either image collection in captive farms or drone deployment during specific periods; however, grassland monitoring requires imagery with both high temporal resolution and high spatial resolution. Wang et al. [8] systematically reviewed estimation methodologies for critical parameters in grassland monitoring, further analyzing their practical applications across ecological context. The mid-infrared camera we choose allows for all-weather image acquisition. Fletcher et al. [9] assembled a mid-infrared camera system to collect plant and soil imagery from both ground-based and aerial platforms, assessing natural resources through analysis of vegetation and soil moisture content. Notwithstanding the demonstrated advantages, mid-infrared cameras present unique challenges in multi-object segmentation. First, mid-infrared images typically suffer from lower resolution and blurred object boundaries, making it difficult to extract clear target contours. Second, long-range imaging results in target objects occupying a small number of pixels in the captured images, further complicating the segmentation process. Additionally, the unpredictable nature of outdoor environments introduces substantial background noises and illumination variations, affecting the accuracy of conventional segmentation algorithms. This necessitates a robust segmentation framework capable of handling the challenges posed by mid-infrared imaging.

To achieve intelligent behavior analysis, we integrate a traditional background subtraction method and a deep learning-based approach. Some traditional background modeling methods include GMM [10], Kernel Density Estimation (KDE) [11], Visual Background Extractor (ViBe) [12], inter-frame difference method [13], Codebook [14], etc. One of the most classic background models is GMM. This method can handle gradual lighting well and can also deal with the intrusion and distance of objects in scenes. However, when the method deals with intermittently moving objects, it is likely to integrate the stationary foreground into the background model. Regarding the problems of GMM in application, Martins et al. [15] proposed a Boosted GMM, which updated the background model through color space classification and dynamic Learning Rate (LR), improving the overall detection accuracy in different scenes while maintaining low complexity. Lu et al. [16] introduced wavelet domain denoising and semi-soft thresholding of foreground objects, which made GMM better adapted to the effects of light variations, noises, and shadows. Liu et al. [17] utilized Kalman filtering to process foreground information, generating and updating the predicted background model, and the results demonstrated that the algorithm accurately detected moving targets in real time. Zhang et al. [18] employed GMM as the core background modeling component and combined it with YOLOv5 for dynamic detection of railway slope falling rocks. Li et al. [19] integrated an adaptive GMM with an enhanced three-frame difference method and further incorporated an illumination compensation algorithm that combines grayscale statistical features with an improved Phong reflection model to effectively address lighting variations and suppress shadows.

Traditional background subtraction methods can already achieve good results, but they remain inadequate for complex scenes. Deep learning-based background subtraction methods can adaptively extract image features by means of supervised learning, obtaining more abstract representations than traditional methods. Braham et al. [20] first applied CNN to background subtraction, utilizing a single grayscale image to analyze complex scenes, training a scene-specific deep background subtraction model to estimate the motion probability of pixel at the center of each window. Lim et al. [21] proposed two encoder-decoder neural networks, Foreground Segmentation Network_M (FgSegNet_M) and Foreground Segmentation Network_S (FgSegNet_S), to generate multiscale feature encoding in different ways. Liu et al. [22] proposed a foreground detection method for multi-scale UNet architecture enhanced by a fusion attention mechanism, which was introduced to direct the network’s focus towards foreground objects and improve its learning ability. Gowda et al. [23] enhanced the foreground segmentation capability of FgSegNet by developing Triple CNN and Transposed Convolutional Neural Networks (TCNN). They integrated a Feature Pooling Module (FPM) to reduce the amount of multi-scale inputs and added up-sampling networks to match the spatial dimensions of abstract image representations to input images. Li et al. [24] proposed a UNet that combines a CNN and the Mamba backbone network for small target segmentation using infrared, demonstrating strong capabilities in extracting local features and modeling global contextual information.

Building on these observations, traditional methods excel in real-time performance, making them suitable for resource-constrained environments. However, they struggle to handle complex scenarios involving occlusions or dynamic illumination changes. Deep learning approaches achieve higher accuracy and overcome traditional limitations through superior feature extraction capabilities. Nevertheless, they require substantial computational resources and extensive training data. Thus, we integrate the strengths of both approaches: the traditional framework provides efficient background modeling, while deep learning enhances feature representation. The above review of background subtraction methods has been summarized in a table, as shown in Table 1.

In this paper, we propose an improved background subtraction method, i.e., Image Timing Features–Gaussian Mixture Model (ITF-GMM), and a deep learning network, i.e., Convolutional-UNet (Con-UNet). After acquiring the surveillance video data, the ITF-GMM algorithm is used to extract the moving objects from image sequences, while the Con-UNet algorithm is applied to compute the segmentation results of foreground targets. Finally, the results of the above two methods are fused for segmenting monitoring objects in mid-infrared images. Our main contributions are as follows. First, regarding algorithm innovation, we introduce an integrated ITF-GMM and Con-UNet for small-scale target detection, specifically addressing the stay-hole problem inside the objects. The stay-hole problem arises due to the intermittent movement of sheep, causing the central areas of flocks to show the same color across frames repeatedly. As a result, the Gaussian Mixture Model (GMM) mistakenly incorporates this color into the model, and foreground pixels matching it are misidentified as background, leading to internal holes in the detection results. Our proposed framework also improves the feature extraction capability of the UNet model, making it more effective for small-target detection with limited training data. Second, as for the application of mid-infrared images, they enable long-distance and all-weather observations [3]. This paper designs a mid-infrared camera monitoring system that facilitates automated law enforcement for grassland grazing regulations. The proposed framework extends the application of mid-infrared imaging for continuous small scale multi-object detection, promoting the digitalization of grassland resource supervision.

In the following sections, Section 2 provides an exhaustive review of existing background/foreground segmentation algorithms. Section 3 details the implementation aspects of proposed method. Section 4 analyzes and discusses the results of the qualitative and quantitative comparisons of our algorithms. Section 5 summarizes the work conducted.

## 2. Methods

### 2.1. Proposed Computational Flow Chart

**Figure 2:**
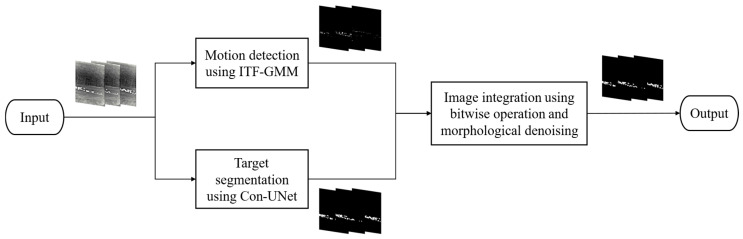
The flowchart of our proposed method.

The flowchart of grassland safety monitoring model is shown in Figure 2, and the main computational processes are as follows. First, mid-infrared images are collected under different weather conditions. Next, the ITF-GMM is initialized to build the background model of grassland, the LR is adjusted using the image content differences of video frames, the number of Gaussian distributions is adjusted using the temporal features, and the moving foreground target is obtained at this stage. Third, the Con-UNet is trained using manually labeled images to extract the features of foreground target, and the foreground object is segmented using the trained network model. Finally, the ITF-GMM and Con-UNet results are fused at the pixel level using bitwise operation to perform morphological denoising of the image. By integrating GMM-based background modeling with a CNN-based segmentation approach, this method combines the strengths of both techniques. On the one hand, the ITF-GMM module is employed to capture frame-level motion patterns, which utilizes temporal information to detect moving foreground objects while preventing the feature extraction network from misclassifying stationary background elements as targets. On the other hand, Con-UNet is introduced to improve spatial detail preservation in small-object segmentation. It extracts target features through the network, allowing for correction of error-prone regions in motion detection. As a result, it can satisfy security monitoring needs.

### 2.2. Key Computational Methods

#### 2.2.1. ITF-GMM

GMM is a background subtraction method used in video target detection which assumes that pixels are independent of each other and that the background color of each pixel can be modeled as a weighted sum of multiple Gaussian distributions [25], each of which represents a background color in image; the weights indicate how often this background color appears in an image. For pixel points in the background, they can be modeled as part of a Gaussian distribution, because their gray values are relatively stable, whereas for pixel points in the foreground, which do not fit the characteristics of Gaussian distribution, the algorithm assigns a dynamic threshold to each pixel at runtime, and a pixel is considered to be foreground if the pixel value in a frame exceeds this threshold. Thus, foreground extraction can be achieved by detecting pixel points outside the Gaussian distribution.

When processing video frame images, it is assumed that the R, G, and B color channels of image pixel points are independent of each other and have the same variance [26]. For a randomly selected pixel *m* at time point *t*, the Gaussian mixture distribution probability density function *p*(*m_t_*) obeys Equations (1)–(3).

(1) $$p\left(m_{t}\right)=\sum_{i=1}^{N}\omega_{i,t}\times \eta \left(m_{t},\mu_{i,t},\tau_{i,t}\right)$$

(2) $$\eta \left(m_{t},\mu_{i,t},\tau_{i,t}\right)=\frac{1}{{\left|\tau_{i,t}\right|}^{\frac{1}{2}}}e^{-\frac{1}{2}{\left(m_{t}-\mu_{i,t}\right)}^{T}{\tau_{i,t}}^{-1}\left(m_{t}-\mu_{i,t}\right)}$$

(3) $$\tau_{i,t}={\sigma_{i,t}}^{2}I$$

where *m_t_* means a selected pixel at time *t*; *N* denotes the total number of Gaussian distributions; *ω_i_*_,*t*_ means the weight of the *i*th Gaussian distribution at time *t*; *η*(*m_t_*, *μ_i_*_,*t*_, *τ_i_*_,*t*_) represents the *i*th Gaussian distribution at time *t*; *μ_i_*_,*t*_ is its mean; *τ_i_*_,*t*_ is its covariance matrix; *σ_i_*_,*t*_ is the variance; and **I** indicates a three-dimensional unit matrix.

A list of Gaussian distributions is firstly initialized for each pixel, each with equal initial weight, i.e., 1/*N*; the covariance matrix is set to unit matrix, and LR is set to default −1. For a new input video frame, each new pixel *m_t_* in the image is compared with the currently existing model according to Equation (4) until a distribution model matching the new pixel value is found.

(4) $$\left|m_{t}-\mu_{i,t-1}\right|\leq 2.5\sigma_{i,t-1}$$

For matching a successful Gaussian distribution model, since the pixel belongs to the background, then the mean and weight are updated based on Equations (5)–(7).

(5) $$\omega_{i,t}=\left(1-\alpha \right)\times \omega_{i,t-1}+\alpha$$

(6) $$\mu_{i,t}=\left(1-\rho \right)\mu_{i,t-1}+\rho m_{t}$$

(7) $${\sigma^{2}}_{i,t}=\left(1-\rho \right){\sigma^{2}}_{i,t-1}+\rho \left(m_{t}-\mu_{i,t-1}\right){\left(m_{t}-\mu_{i,t-1}\right)}^{T}$$

where *ω_i_*_,*t*_ denotes the weights of the updated Gaussian distribution and *α* represents the LR; then the weights of each model are normalized.

The mean *μ* and standard deviation *σ* of the unmatched patterns are unchanged, and the weights are updated according to Equation (8).

(8) $$\omega_{i,t}=\left(1-\alpha \right)\omega_{i,t-1}$$

To suppress the impact of noise and non-background pixels, it is typically necessary to compute a weighted average of Gaussian distribution weights. The models are arranged in descending order according to *ω*/*σ*^2^, and the models with large weights and small standard deviation are ranked in the first place. After sorting, the first *B* Gaussian distributions that conform to Equation (9) are regarded as the background model, thereby determining the value of *N*. The parameter *T* denotes the minimum proportion of background occupied by background; it is a threshold with a value range of 0.5 to 1.0.

(9) $$B=\arg\left\{\underset{b}{\min}\left(\sum_{n=1}^{b}\omega_{i,t}\geq T\right)\right\}$$

Due to the small number of pixels occupied by small-scale targets, which are usually characterized by intermittent movement, the foreground pixels exist for a long time and are integrated into the background model when using the GMM for background modeling; the segmentation results in the appearance of the stay-hole problem. To address the problems above, this paper proposes an ITF-GMM, which utilizes the temporal characteristics of images and optimizes the effect of GMM by dynamically adjusting the LR of GMM and the number of Gaussian distributions.

- Dynamic adjustment of LR

LR is an important parameter to control the speed of model updating. Too high of an LR will lead to model overfitting, and too low of an LR will cause failure of adaptation to scene changes. In this paper, we start from the reason for slow change between neighboring frames of surveillance data and adaptively adjust the LR according to the variation between two neighboring images. In this paper, Structural Similarity Measure (SSIM) is chosen to evaluate the image similarity [27], which takes into account the effects of brightness, contrast, and structure. Assuming that *j* denotes the first frame of image data and *k* denotes the second frame of image data, the SSIM calculation formula is shown in Equation (10).

(10) $$X_{SSIM}=\frac{\left(2\mu_{j}\mu_{k}+C_{1}\right)\left(2\sigma_{jk}+C_{2}\right)}{\left({\mu_{j}}^{2}+{\mu_{k}}^{2}+C_{1}\right)\left({\sigma_{j}}^{2}+{\sigma_{k}}^{2}+C_{2}\right)}$$

where *μ_j_* and *μ_k_* represent the mean gray scale values of images *j* and *k*, respectively; *σ_j_*^2^ and *σ_k_*^2^ are the variance of corresponding images, respectively; *σ_jk_* means the covariance; *C*_1_ and *C*_2_ are constants, avoiding a denominator of zero.

SSIM employs global images as its reference framework, while grassland monitoring data are characterized by a small number of pixels and a concentrated region of foreground targets. If the whole image is calculated, it will increase the amount of unnecessary resource consumption and also introduce disturbances such as tree branch jitter and lighting changes into it. Therefore, we firstly extract the Region of Interest (ROI) from the target set. To construct the ROI, we initially detect foreground targets in the first 100 frames and define a bounding box that tightly encloses all detections. This box is then used as the fixed ROI throughout subsequent processing. This strategy avoids manual intervention while ensuring the ROI adapts to actual foreground dynamics. A properly selected ROI reduces background clutter interference and enables SSIM-based LR adjustment to better reflect object motion. If the ROI selected is too small, it may lead to inaccurate detection results. Conversely, if the ROI selected is too large, it may reduce the efficiency of the algorithm. Within this region, the SSIM values of images in two neighboring frames are calculated. If the two frames are similar, the SSIM has a value close to 1, which indicates that the target is moving slowly and the LR needs to be reduced, preventing the foreground target from integrating into the background. On the contrary, if the two frames are less similar, the SSIM has a value close to 0, which indicates that the scene has changed drastically, and the LR needs to be increased to adapt to the new scene. After the above analysis, it is known that the values of SSIM and LR are negatively correlated. The formula of LR is obtained after experimental analysis, as shown in Equation (11).

(11) $$LearningRate=\frac{1-SSIM}{80}$$

- Dynamic adjustment of Gaussian distribution numbers

The number of Gaussian components can determine the complexity and sensitivity of the whole model. By adaptively learning the number of Gaussian distributions, complex background scenes can be modeled more accurately. Outside the ROI, the scene is more stable, and the number of Gaussian distributions is set to 2. Inside the ROI, for each individual pixel, a grayscale histogram of the time series is constructed. Peaks in these histograms indicate grayscale values with prolonged temporal persistence and stability, which significantly influence the background model and should be modeled as Gaussian distributions. First, our method finds the number of peaks in the time series histogram by Equation (12), then it filters the peaks according to the size of the threshold. If the distance between two neighboring peaks is less than the threshold, these two histogram peaks will be regarded as a single peak; otherwise, the histogram peak is retained. The threshold used for histogram peak filtering is set to 30 based on empirical validation. This value effectively eliminates insignificant peaks caused by noise, while preserving genuine intensity modes contributing to Gaussian components. Finally, our model sets the number of Gaussian distributions to the corresponding number of peaks, as shown in Equation (13).

(12) $$\left\{\begin{array}{c} h\left(i+1\right)-h\left(i\right)<0\\ h\left(i\right)-h\left(i-1\right)<0 \end{array}\right.$$

(13) $$N\left(x_{t}\right)=P\left(t\right)$$

where *h*(*i*) denotes the number of pixel values at point *i*, 0 < *i* < 255; *N*(*x_t_*) is the number of Gaussian distributions of pixel *x* at moment *t*; and *P*(*t*) represents the number of peaks in the histogram at moment *t*.

In summary, the pseudocode that describes the procedure of ITF-GMM is shown below (Algorithm 1).

**Algorithm 1:** ITF-GMM**1. Input** Video frame sequence**2. Output** Foreground masks for each frame**3. Initialization** **for** each pixel *x* **do**      Initialize *N* Gaussian distributions **end for****4. Procedure process** **for** each frame **do**      Extract ROI      Compute dynamic LR via Equation (11) in ROI      Compute dynamic Gaussian components via Equations (12)–(13) in ROI      **for** each pixel *x_t_* **do**            Compare *x_t_* with existing model via Equation (4)            **if** a match is found **then**                  Mark *x_t_* as background                  Update parameter via Equations (5)–(7)            **else if** no match **then**                  Mark *x_t_* as foreground                  Update weights via Equation (8)            **end if**            **if** *x_t_* ∈ ROI **then**                  update LR and N            **else**                  N = 2            **end if**            Normalize all weights to ensure sum is 1 and *N* satisfies Equation (9)      **end for** **end for**

GMM is able to continuously update the model according to video image sequence, distinguishing the moving foreground from the stationary background. This allows it to monitor target movement in complex scenes, with the advantage of considering timing information between video frames. However, the grassland environment is variable and has many influencing factors, so there is still a stay-hole problem and poor robustness with the above improvements. Therefore, a CNN-based segmentation method is used to learn the target’s contour, posture, and other features, establishing a segmentation model of grassland monitoring target. This method lacks consideration of the temporal correlation between frames when dealing with video images, which are considered to be independent of each other and relatively complicated for computation. The combination of two methods can effectively reduce the dependence of the experiment on lighting conditions and overcome the influence of the grassland environment.

#### 2.2.2. Con-UNet

**Figure 3:**
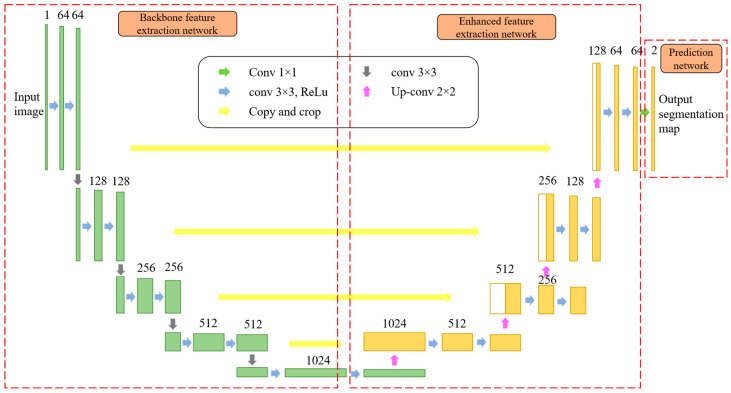
Proposed Con-UNet network structure.

CNN is a feed-forward neural network that uses convolutional computation to extract features from the original input data. By learning the hierarchical representation, CNN has the ability to sort input information with high accuracy, and it is applied in classification tasks on a large scale; the output is class labeling of the whole image. In this paper, the infrared image data sample size is small, and the network cannot be trained using a large number of images. After comprehensive analysis, a UNet segmentation network [28] was selected, which is a fully convolutional network that can achieve pixel-level classification. The output is the class of each pixel point, and pixels of different categories will display as different colors. In addition, the UNet network does not need a large training set to obtain accurate segmentation results. It has a relatively short training time, a simple structure, and lower parameter requirements than other networks. In order to extract as many features as possible with limited data and improve the feature extraction capability of the UNet network, Con-UNet is proposed, and the network structure is shown in Figure 3. The key innovation of Con-UNet lies in replacing the traditional maximum pooling layer with convolutional subsampling layers. This modification preserves more spatial information—particularly crucial for small and indistinct targets—thereby improving the overall representation ability and adaptability of the model.

Con-UNet is realized by three modules: convolution, subsampled, and upsampling. In Figure 3, the left is the backbone feature extraction network, which has the same number of feature channels and ReLU activation functions within the convolutional layer. The convolutional panel is a 3 × 3 convolution with a step size of 1, a padding of 1, and a padding mode of reflect. A symmetric element is taken for each point to ensure that the entire graph has features. Since maximum pooling has the ability to extract features and will miss many features, the subsampling block does not adopt the 2 × 2 maximum pooling layer in the original network, but uses a 3 × 3 convolution with a stride of 2 for subsampling, and the padding mode of reflect. The right network is an enhanced feature fusion network, which uses the feature maps from upsampling to conduct concatenated operation with the left feature map. The upsampling node consists of two 3 × 3 convolutions and one 2 × 2 deconvolution. To prevent holes after convolution, upsampling uses the nearest interpolation. The output layer is activated using Sigmoid. Although the final output is a color image, this paper is a binary classification problem for each pixel, and it only needs to classify each pixel. Finally, there is a prediction network that generates the segmentation results. The network is characterized by jump connections through symmetric layers of the encoding part and decoding part, which enhances the image segmentation capability of network.

## 3. Results

### 3.1. Experimental Environment

A large number of experimental studies have been carried out to address the research problems in this paper. The simulation experiments in this paper are developed in Python, the operating system used for experiments is Windows 10, the CPU model is Intel(R) Core(TM) i7-10700K CPU @ 3.80 GHz, the graphics card is NVIDIA GeForce RTX 3090, the computer is working by solo display during the training, the development environment is Python 3.8, and the deep learning environment is OpenCV 4.6.0.

### 3.2. Dataset

**Figure 4:**
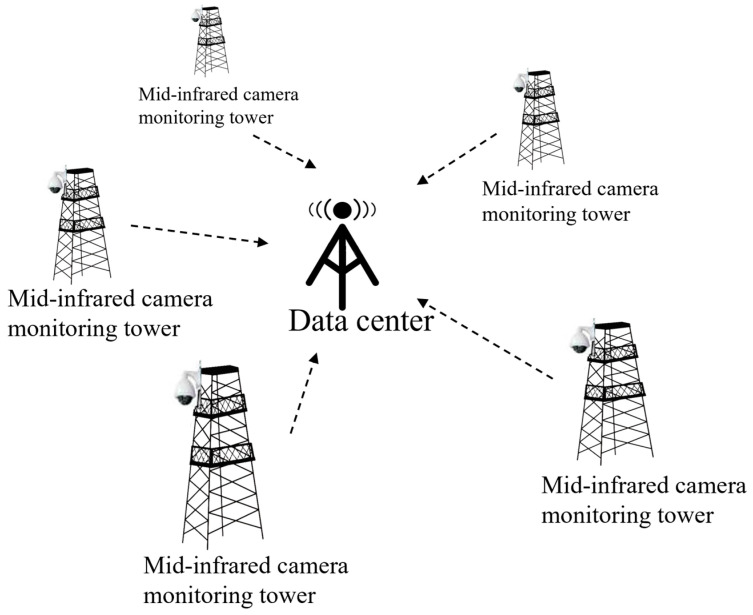
Mid-infrared camera tower network system.

**Figure 5:**
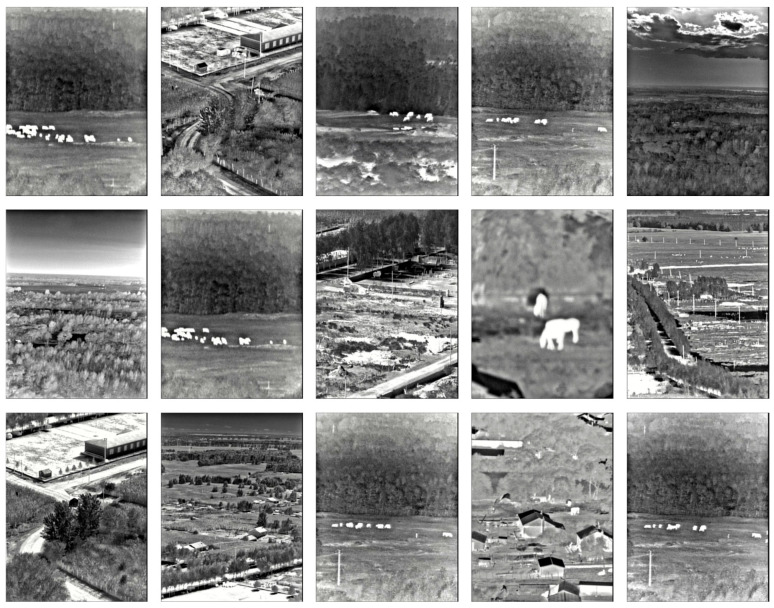
Examples of some mid-infrared images.

The experimental data used in this paper are collected in Inner Mongolia, China. We design a network system of observation stations with mid-infrared camera tower clusters in the grassland ecosystem; the schematic diagram is shown in Figure 4. The shooting distance is 5.0–10.0 km roughly, and a mid-infrared camera with a waveband range of 2.5–25.0 μm [29] is set up on tower to obtain all-weather data information from the grassland. Each instrument can transmit monitoring data to the data center for subsequent processing. An example of the acquired images is shown in Figure 5; it is captured using our own mid-infrared imaging system during different times of the day. All the training and detection tasks are also carried out based on the self-built dataset. After data selection, a total of 5000 images are obtained. To ensure effective model training and validation, we label 129 images and then divide these data into a training set and a validation set at a ratio of 8:2.

### 3.3. Evaluation of Proposed Computational Methods

**Figure 6:**
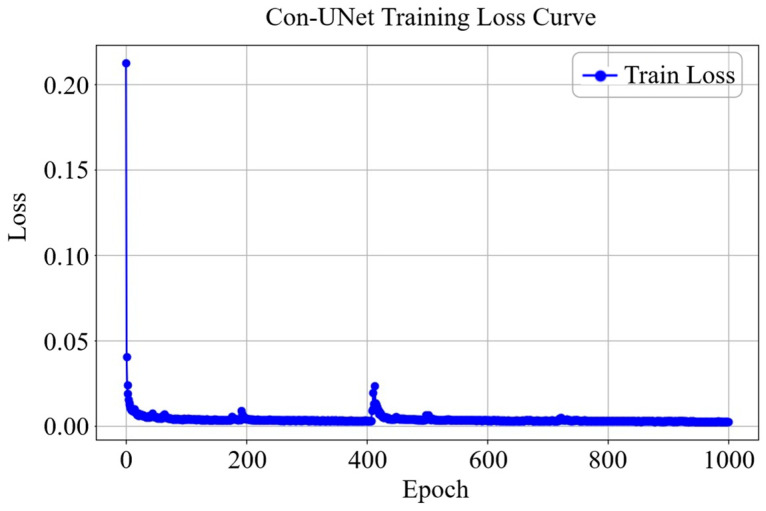
Learning curves through Con-UNet training.

**Figure 7:**
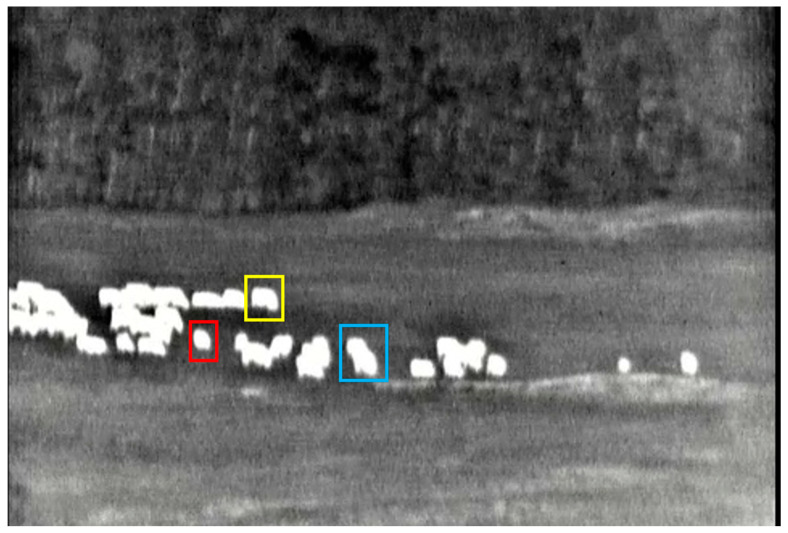
Sheep labeling graphical representation. The red box represents individual sheep facing the camera; the yellow box means individual sheep facing away from the camera; the blue box shows overlapping groups of sheep.

**Figure 8:**
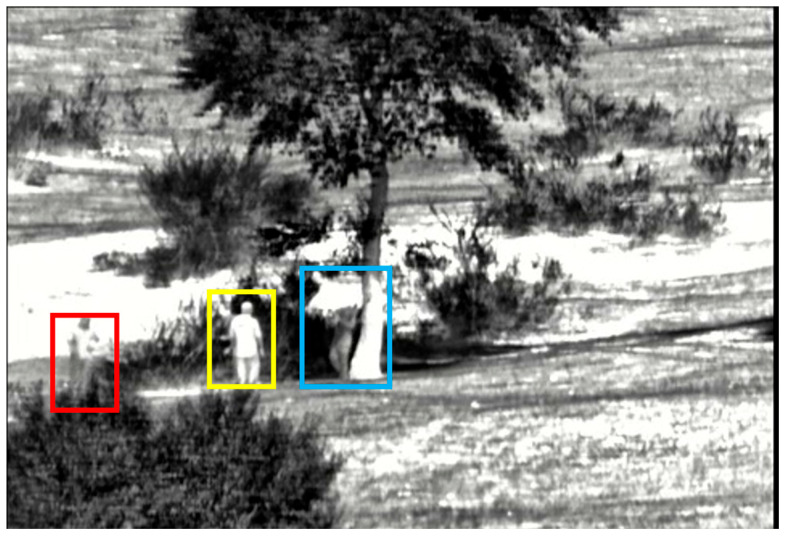
Crowd labeling graphical representation. The red box indicates the front of the person; the yellow box represents the back of the person; the blue box means the side of the person.

In order to avoid the influence of different parameters on the experimental results, both the UNet model and the proposed Con-UNet model are trained using the same dataset and hyperparameter settings. All images, originally sized 480 × 638, are uniformly resized to 256 × 256 before being input into the network. The segmentation models are implemented using the PyTorch 1.2.0 deep learning framework, trained for 1000 epochs with a batch size of 2. The optimizer used is Adam, and the loss function is cross-entropy. The learning curves through training are shown in Figure 6. The trained images are labeled using Labelme [30]. We define facing the camera as the front. The labeled sheep include a single sheep with its head down grazing on the front side, as shown in the red box in Figure 7; a single sheep walking on the side, as shown in the yellow box in Figure 7; two sheep with overlapping portions, as shown in the blue box in Figure 7; and flocks, which contain the majority of the possible shapes of grassland sheep. The labeled people include the front of a person, as shown in the red box in Figure 8; the back of a person, as shown in the yellow box in Figure 8; and the side of a person, as shown in the blue box in Figure 8, encompassing most of the possible poses of a person.

**Figure 9:**
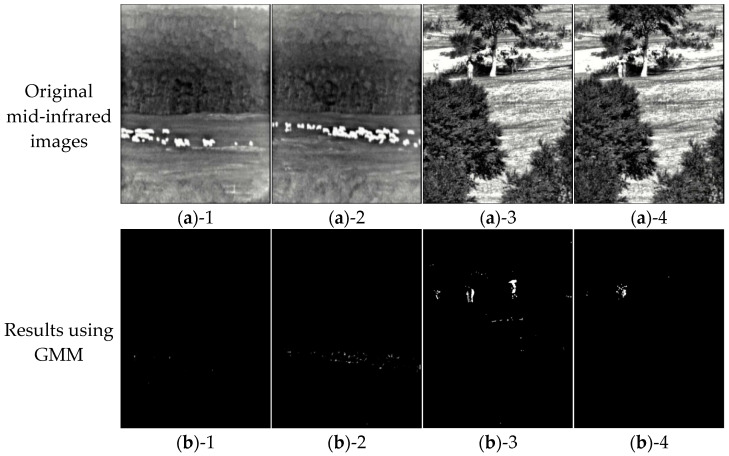

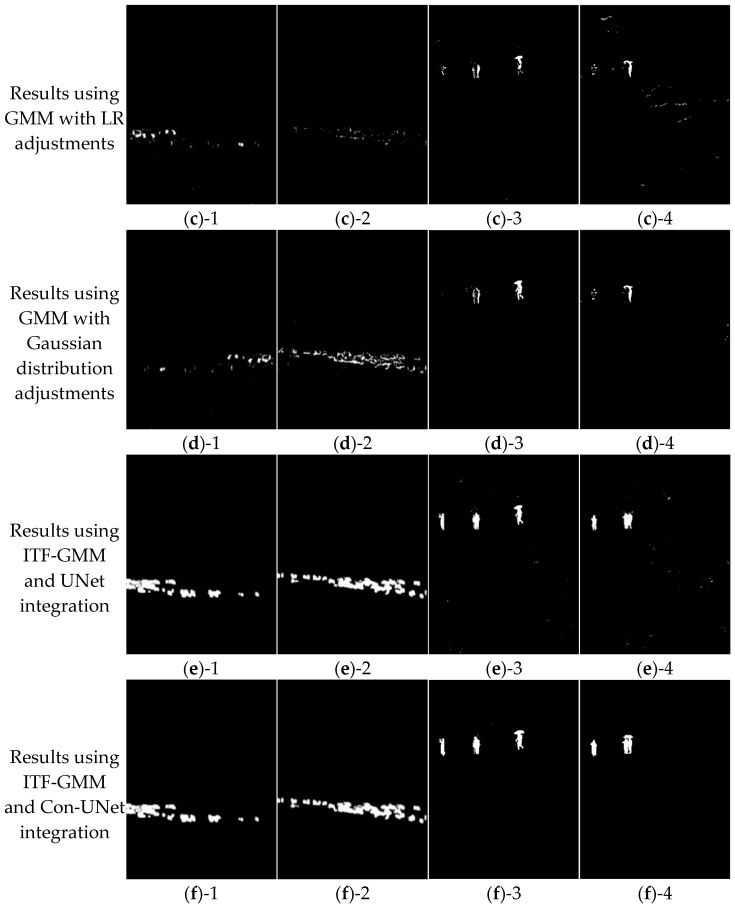
Segmentation results using different computational strategies. (**a**)-1–(**a**)-4 are the original mid-infrared images; (**b**)-1–(**b**)-4 are the results using GMM; (**c**)-1–(**c**)-4 are the results using GMM with LR adjustments; (**d**)-1–(**d**)-4 are the results using GMM with Gaussian distributions adjustments; (**e**)-1–(**e**)-4 are the results using ITF-GMM and UNet integration; and (**f**)-1–(**f**)-4 are the results using ITF-GMM and Con-UNet integration.

In order to confirm the effectiveness of modified methods for grassland small-scale sheep and crowd recognition, on the basis of GMM, only one improvement method is added at each step, and the influence of each approach on the original algorithm is verified. Five experiments are set up. Experiment 1 denotes evaluation of the original GMM. Experiment 2 means evaluation of the GMM calculation with adjustment of the LR based on image temporal features. Experiment 3 indicates evaluation of GMM that adjusts the LR and the number of Gaussian distributions, i.e., ITF-GMM. Experiment 4 represents integration evaluation of ITF-GMM and UNet. Experiment 5 is the integration evaluation of ITF-GMM and Con-UNet. The segmentation results of the experiments are shown in Figure 9. In order to evaluate the detection accuracy of the proposed algorithm objectively, we also use four metrics: accuracy, precision, intersection over union (IOU), and recall [31]. At the same time, in order to evaluate the real-time performance of the algorithm, we calculated the interfering time and Floating Point Operations (FLOPs) [32], which are calculated as shown in Table 2.

As can be seen from Table 2, the improved model optimizes various metrics compared to the original GMM, where the value of accuracy is consistently higher, indicating that more than 90.0% of the foreground pixels recognized by the algorithm are indeed the examined target, and a small percentage of them are background but are mistaken for the target. For the other metrics, GMM with LR adjustments increases IOU from 0.0172 to 0.0724 and recall from 0.0174 to 0.0777, but precision decreases from 0.6307 to 0.5169. This may be attributed to the fact that the LR can be better adapted to scene changes, allowing targets to be better detected. The Experiment 3 model increases precision from 0.5169 to 0.7562, IOU from 0.0724 to 0.2448, and recall from 0.0777 to 0.2657, indicating that adjusting the number of Gaussian distributions creates a more realistic background model. The Experiment 4 model improves precision from 0.7562 to 0.9021, IOU from 0.2448 to 0.9347, and recall from 0.2657 to 0.9136, showing UNet’s powerful feature extraction ability. The Experiment 5 model advances precision from 0.9021 to 0.9613, IOU from 0.9347 to 0.9461, and recall from 0.9136 to 0.9702. In terms of the real-time performance of the algorithm, the interfering frame rates for Experiment 1 to Experiment 5 are 22.33 fps, 21.07 fps, 17.56 fps, 9.15 fps, and 7.26 fps, respectively. Although the integration of Con-UNet increases runtime compared to GMM, it significantly improves the detection accuracy and remains acceptable for real-time applications. FLOPs quantify the total number of floating-point multiplications and additions required by a computing device during execution of a model. In general, CNNs require such operations, while the computational complexity of GMM is much lower. Therefore, we calculate the FLOPs of UNet and Con-UNet, which are 60.24 and 63.44, respectively. This indicates that the modules we proposed will not significantly increase computational complexity compared to UNet. From the subjective results and objective indices combined, ITF-GMM shows superior performance in small-scale multi-objective segmentation.

In order to further validate the superiority of our method, we conduct comparative experiments on our mid-infrared video dataset and consider different background subtraction methods. The algorithms used for comparative evaluation are: inter-frame difference, ViBe, KNN, background subtraction in the literature [33], Recurrent Residual UNet (R2UNet) [34], and Attention UNet (Att UNet) [35]. The inter-frame difference method obtains moving targets by performing a differencing operation on two consecutive frames; when there is target motion in the monitoring scene, there will be more obvious differences between two adjacent frames. By subtracting two frames of images, the absolute value of pixel difference at the corresponding position can be obtained, and it can be judged whether it is greater than a certain threshold; then the motion characteristics can be analyzed. ViBe uses neighborhood pixels to create a background model and detects the objective by comparing the background model with the current input pixel value. This algorithm has a mechanism to update over time, which makes the background model change with variations of moving objects in the image range. The basic idea of KNN is to perform a parameter-free probability density estimation of the historical pixel values of each pixel in the time domain, to determine whether these historical pixel values belong to the background or foreground class, and when there is a new input of image frames. The KNN algorithm compares each pixel in the image with the historical pixel value of point and determines whether or not the pixel is a foreground object based on a specified threshold. R2UNet extends the UNet by incorporating a Recurrent Residual Convolutional Neural Network (RRCNN), which leverages dual-layer recurrent convolution and residual connections to enhance feature extraction, thereby improving the model’s overall feature representation capability. Att UNet integrates an Attention Block, enabling the network to focus on critical feature regions while suppressing irrelevant features.

**Figure 10:**
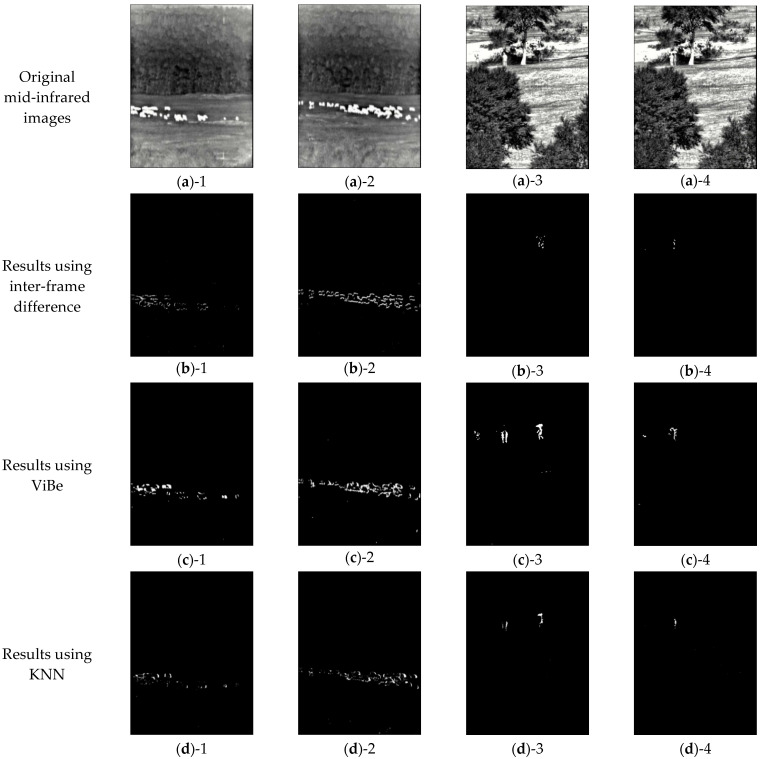

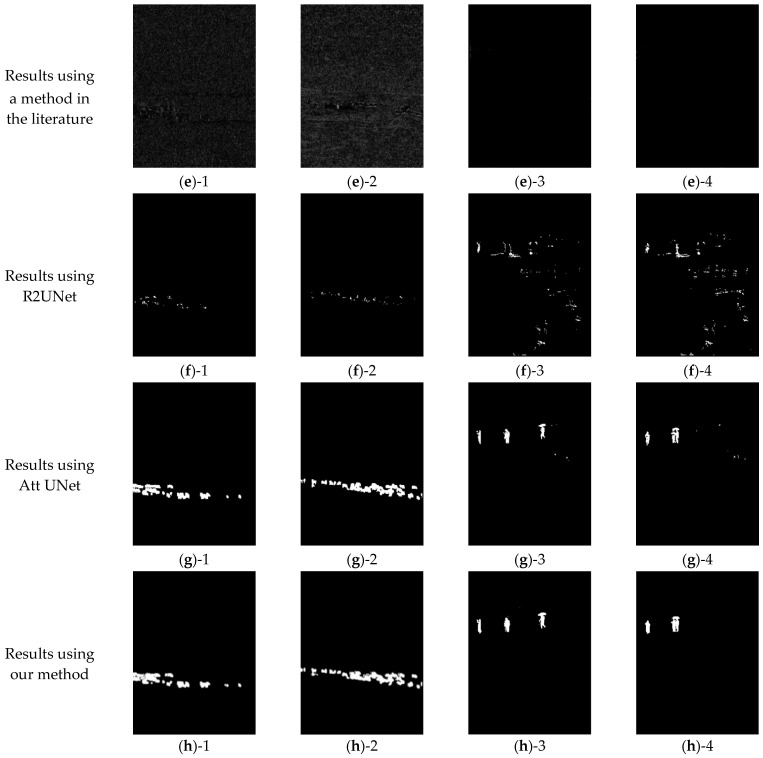
Segmentation results using different algorithms. (**a**)-1–(**a**)-4 are the original mid-infrared images; (**b**)-1–(**b**)-4 are the results using inter-frame difference; (**c**)-1–(**c**)-4 are the results using ViBe; (**d**)-1–(**d**)-4 are the results using KNN; (**e**)-1–(**e**)-4 are the results using a method in the literature [33]; (**f**)-1–(**f**)-4 are the results using R2UNet; (**g**)-1–(**g**)-4 are the results using Att UNet; and (**h**)-1–(**h**)-4 are the results using our method.

In order to visualize the difference in background subtraction performance between ours and other methods, we visualize the segmentation results of above algorithms, as shown in Figure 10.

Figure 10 shows the segmentation results obtained by different algorithms. In each row, the results for the flock and the crowd are shown from left to right, respectively. Figure 10a represents the original mid-infrared image, while Figure 10b–h compare the results of different models. Among them, Figure 10b shows the results of the inter-frame difference method, which can obtain the overall motion contour of targets but has a serious stay-hole problem. Figure 10c demonstrates the results of ViBe, which improves the stay-hole problem, although the target external contour is not as clear and complete as in Figure 10b. Figure 10d indicates the results of the KNN, which exhibit clearer external contours but retain a severe stay-hole problem. Figure 10e presents the results of a method in the literature [33], which is affected by various interference factors, leading to much poorer performance than the other algorithms. Figure 10f shows the results of R2UNet, which fails to detect the target objects completely and introduces additional interference. Figure 10g exhibits the results of Att UNet, which achieves relatively good detection performance; however, the edge transitions appear unnatural, exhibiting a jagged effect. Figure 10h shows our results, which can distinguish between foreground and background interference, obtain clear and complete foreground images of sheep and crowds, and visualize the crowd with natural transitions in finer detail. It can be seen that Figure 10b–g can eliminate the interference of lighting, background, etc., and obtain moving foreground objects, although there is a stay-hole problem. The objective evaluation of the results in Figure 10 are calculated for each algorithm and shown in Table 3.

As can be seen from Table 3, the objective metrics and subjective images show the same effect. For these metrics, smaller training time indicates better performance, while larger values are preferred for all other metrics. The accuracy of the method in [33] is 0.9873, but the other three indicators of precision, IOU, and recall are all 0.0, which shows that the proportion of target pixels predicted correctly is high, the proportion of background pixels mistaken for targets is low, but the number of predicted target pixels is small, and most targets are incorrectly detected as background. Compared with inter-frame difference, ViBe, KNN, R2UNet, and Att UNet, the precision of the proposed model is changed from 0.5939, 0.5748, 0.5180, 0.9985, and 0.9428 to 0.9692, respectively. Although the precision value is slightly lower than that of R2UNet, the difference is marginal. Accuracy is improved from 0.9871, 0.9827, 0.9875, 0.9947, and 0.9895 to 0.9987, respectively. IOU is increased from 0.0954, 0.2112, 0.0506, 0.0184, and 0.9131 to 0.9481, respectively. Other algorithms’ accuracy is higher, but IOU still rises more because there are many pixels that are targets but are misidentified as background. Recall advances from 0.1020, 0.2503, 0.0531, 0.0185, and 0.9626 to 0.9775, respectively. The reason for the much higher values is that the other algorithms obtain a small percentage of all the target pixels in the foreground pixels and have a high rate of missed detection. In addition, to meet the real-time requirements of application, training time and interfering time are introduced. The surveillance video has a frame rate of 10 fps, while the interfering frame rates of R2UNet, Att UNet, and our model are 6.46 fps, 7.47 fps, and 7.26 fps, respectively. R2UNet exhibits poor real-time performance, whereas Att UNet performs better. However, considering both detection accuracy and real-time performance, the proposed model outperforms the other models.

## 4. Discussions and Interpretive Analyses

At present, China is committed to constructing an ecological civilization and ecological environmental protection, strengthening the study of relevant laws and regulations and policy systems, intensifying judicial protection of the ecological environment, and actively carrying out publicity and education about environmental protection. In particular, it has vigorously promoted the digital construction of an ecological environment and continuously improved policy systems conducive to in-depth integration of digital technology and the construction of an ecological civilization [36]. This paper addresses the problem of monitoring grassland violations, introduces high-tech means such as mid-infrared cameras and intelligent algorithms, and proposes new solutions for the protection and management of grassland resources. During a period of grazing moratorium, if there are flocks or crowds entering, this indicates the emergence of management violations, which need to be dealt with in time. The method proposed in this paper reduces dependence on manual labor and improves the efficiency and technology level. In order to further explore the impact of other influencing factors on the algorithm’s operational results, two supplementary experiments are conducted to test the robustness of our algorithm.

In order to test whether significant imparity exists in the image qualities acquired at different times and affects the segmentation results, we take sheep targets as an example, selecting sheep data acquired in the morning and the afternoon for segmentation algorithm evaluation. Here, we determine whether there is a difference in image quality firstly, and then calculate the segmentation results and analyze them.

There is no reference when estimating image quality, so we choose a reference-free image quality evaluation index. Regarding reference-free image metrics, clarity of image is an important indicator, which can better correspond to the subjective cognition of people. We use several commonly used and representative sharpness algorithms for calculation. The Brenner gradient function, Roberts function, and standard deviation of the mean difference squared (SMD2) are considered [37]; the larger the result, the clearer the image is. The Brenner gradient function is the simplest gradient evaluation function, which simply calculates the square of the grayscale difference of two neighboring pixels. The Roberts function uses the difference between gray values of pixel points in a diagonal direction; the sum of cross-subtraction squared gray values of four adjacent pixel points is applied as the gradient value of each pixel point, and the gradient values of all pixels are summed up as the value of sharpness evaluation function. The SMD2 function multiplies the difference of two grays in each pixel field and then sums it pixel by pixel. The image is the sharpest and has the highest number of high-frequency components when it is completely in focus. The sharply focused image has a larger gray level difference than the blurred image. The formulas for these evaluation indexes are shown in Equations (14)–(16).

(14) $$D_{B}\left(f\right)=\sum_{y}{\sum_{x}\left|f\left(x+2,y\right)-f\left(x,y\right)\right|}^{2}$$

(15) $$D_{R}\left(f\right)=\sum_{x}\sum_{y}\left\{{\left[f\left(x+1,y+1\right)-f\left(x,y\right)\right]}^{2}+{\left[f\left(x+1,y\right)-f\left(x,y+1\right)\right]}^{2}\right\}$$

(16) $$D_{S}\left(f\right)=\sum_{y}\sum_{x}\left|f\left(x,y\right)-f\left(x+1,y\right)\right|\times \left|f\left(x,y\right)-f\left(x,y+1\right)\right|$$

where *D*_B_(*f*) denotes the result of the image quality calculation using the Brenner gradient function; *D*_R_(*f*) represents the result of the image quality calculation using the Roberts function; *D*_S_(*f*) represents the result of the image quality calculation using the SMD2 function; and *f*(*x*, *y*) is the gray value of corresponding pixel point (*x*, *y*).

The results of the image quality calculation are shown in Table 4, where Img1 and Img2 are the images acquired in the morning and Img3 and Img4 are the images collected in the afternoon.

The text continues here (Figure 2 and Table 3). As can be seen from Table 4, the image quality acquired in the same time period is approximately the same, and the image quality metrics collected in different time periods are slightly different. Taking Img1 and Img3 as examples, if we take Img3, which has a clearer image quality, as a benchmark, we can see that the relative difference for the Brenner metric is 0.2821, with the calculation method being (112,356,710–80,664,844)/112,356,710. In a similar way, the relative difference for the Roberts metric is 0.2476, and the relative difference for the SMD2 metric is 0.3588. One possible reason for the difference in image quality is that the cloud thickness varies during the day, and the energy radiated from the sun to the ground through clouds is also different, resulting in slight differences in the final images. In addition, from sunrise to sunset, the angle of sun irradiation on the ground is different, and the radiated energy may also be different. In short, the mid-infrared camera detects the infrared radiation of targets [38], and the different radiation energy of the object may have a slight effect on the image.

**Figure 11:**
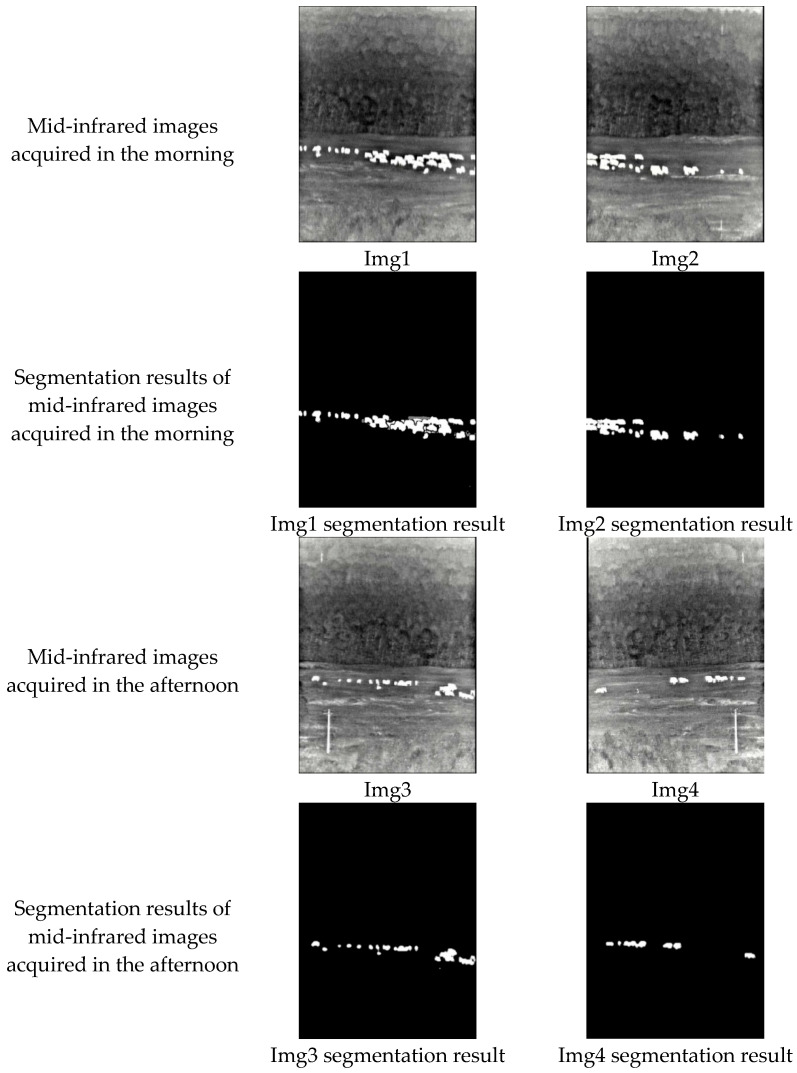
Segmentation results of different quality images.

In order to explore whether the different quality of an image has an influence on the segmentation effect, we also use data acquired at different times to conduct experiments. The results are shown in Figure 11, and the evaluation metrics are shown in Table 5.

As can be seen in Figure 11, at a subjective level, Img1 to Img4 are all able to segment the sheep well without being affected by differences in image quality, excluding interference from background such as forest and pasture, and obtaining a clear foreground image. According to Table 5, it can be seen that, from the level of objective metrics, Img1 to Img4 all achieve a better segmentation effect, and the precision, accuracy, IOU, and recall all basically reach above 0.9, which indicates that the algorithm can accurately and comprehensively recognize the target. Additionally, we can observe that, although Img3 has a relatively high image quality, the presence of partially overlapping sheep results in lower segmentation performance, especially in terms of precision and IOU. This illustrates that segmentation performance may be more sensitive to scene structure than absolute sharpness. Poor image quality may have an impact on the feature extraction stage with fuzzy edges, but motion detection can obtain a complete outline of an object’s movement, and the two complement each other to reduce the adverse effects produced by image quality. Based on the above analysis, it can be seen that the mid-infrared images acquired during the day are different, but they have little effect on the segmentation results. This not only proves the ability of the mid-infrared camera to work around the clock, but also demonstrates that the algorithm in this paper can resist the influence of image clarity.

**Figure 12:**
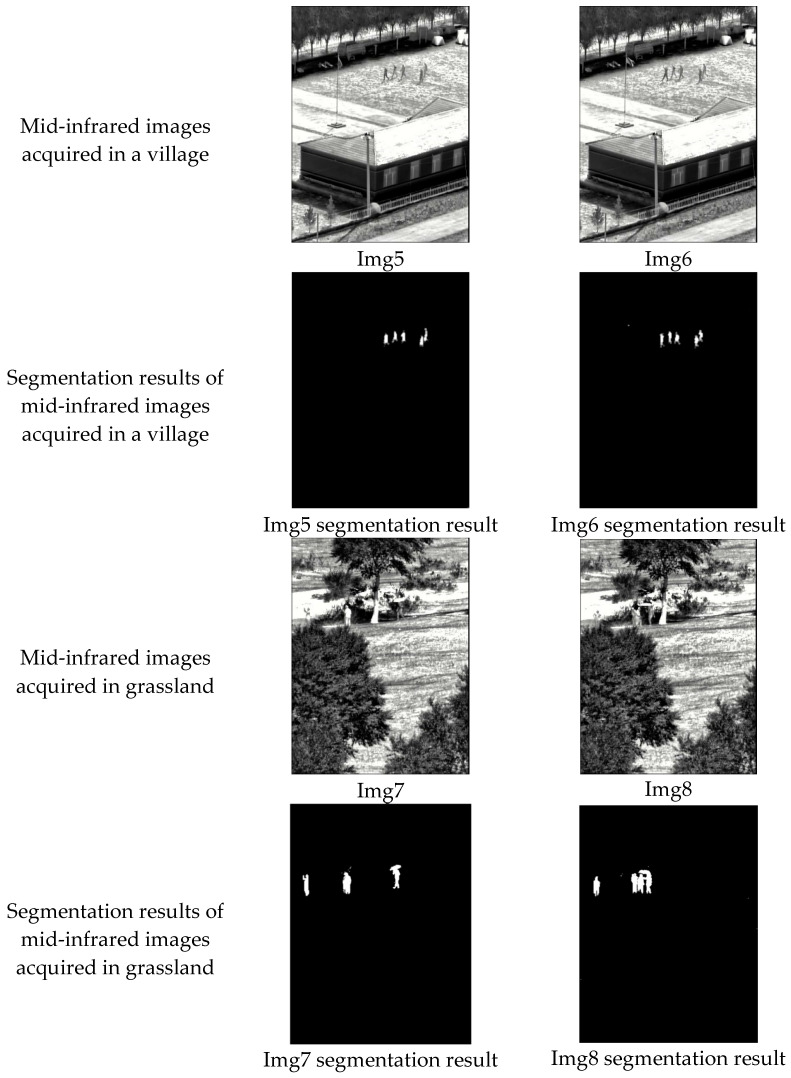
Segmentation results of different scenes.

In order to test the segmentation ability of the algorithm for the same type of target in different scenes, we take the crowd target as an example and carry out experiments with a grassland background or a village background. The experimental results are shown in Figure 12, and the experimental metrics are shown in Table 6.

From a subjective evaluation point of view, Img5 to Img6 exclude the interference of houses, flags, and other backgrounds in the village scene and obtain a clear foreground image of the crowd; Img7 to Img8 exclude the interference of trees, large reflective ground, and other backgrounds in the grassland scene and separate the crowd from the background. From an objective evaluation, the precision, accuracy, IOU, and recall of Img5 to Img8 reach more than 0.9, and the values of the indices of different scenes are not clearly different. The above data show that our algorithm can extract crowd features, even if other moving objects are introduced in the first step of motion detection. When using the Con-UNet network, the desired crowd features can be accurately extracted without being interfered with by backgrounds such as fluttering flags. Based on the above analysis, our algorithm has the ability to exclude interference from different scenes and detect the same type of target, and can be migrated to other scenes for use with high robustness.

The advantage of our algorithm is that it uses a combination of traditional background-subtraction-based and deep learning-based methods to detect and segment small-scale multi-targets in grassland. From the experimental results, our algorithm can obtain clear and accurate segmented images. According to a comprehensive evaluation of various quantitative metrics, our method achieves a low leakage rate and false detection rate. In terms of algorithm migration, our approach has good robustness for different image qualities; for the same type of target, it can exclude the interference of different backgrounds and achieve good results. Additionally, the proposed method is not limited to grassland monitoring and can be extended to other pixel-level segmentation tasks, further demonstrating its generalizability. Finally, from the perspective of engineering application, we design an economical and reliable data acquisition system, providing an effective solution for grassland video supervision.

## 5. Conclusions

In this paper, we propose a detection algorithm that integrates ITF-GMM and Con-UNet, which has a better effect for mid-infrared images. The ITF-GMM highlights the foreground targets by dynamically adjusting the LR and optimizing the number of Gaussian distributions. First, the ROI is obtained. Within the ROI, SSIM is applied to compute the difference between two neighboring frames. Based on the calculation results, the motion speed of the foreground objects is analyzed. If the movement is slow, we reduce the LR; otherwise, we increase the LR. For the number of Gaussian distributions, within the ROI, a time series histogram is computed for each pixel, and the number of Gaussian distributions is increased or decreased according to the number of histogram peaks. The Con-UNet replaces the traditional maximum pooling layer with convolution in the sampling session, enhancing feature extraction capability. The trained Con-UNet is used to segment the image sequence, and its segmentation results are fused with ITF-GMM. Finally, a morphological operation is performed to eliminate image noise. Our method combines the advantages of traditional methods and deep learning, achieves high segmentation precision (96.92%), and maintains real-time performance (7.26 fps), while demonstrating robustness across different backgrounds. However, our algorithm also has certain shortcomings. The current model is trained with a limited number of labeled samples and tested within a specific domain. This may affect its ability to generalize to unseen targets or environments. Future work will aim to validate the approach on different species and geographic scenes to assess its generalizability. In addition, our method depends on labeled data for new target categories. When detecting previously unseen object types, manual annotation and network model training are required during the pre-monitoring stage. In the future, we plan to enhance the network architecture and improve its feature extraction capability. It is expected that features can be better extracted from smaller training datasets, thus reducing the pressure of manual annotation. Furthermore, we will investigate domain adaptation techniques or semi-supervised learning frameworks to reduce dependency on fully labeled datasets.

## Figures and Tables

**Table 1:** A summary table of background subtraction methods.

Method	Representative Methods	Advantages	Disadvantages
Traditional background subtraction method	GMM, KDE, ViBe, etc.	High real-time performance	Poor performance in complex scenarios
Deep learning-based method	CNN, UNet, etc.	Strong feature extraction capability	High demand for computing resources

**Table 2:** Evaluation metric comparisons using different computational strategies *.

	Experiment 1	Experiment 2	Experiment 3	Experiment 4	Experiment 5
GMM	√	√	√	√	√
GMM with LR adjustments	-	√	√	√	√
GMM with *N* adjustments	-	-	√	√	√
UNet	-	-	-	√	-
Con-UNet	-	-	-	-	√
Precision	0.6307	0.5169	0.7562	0.9021	0.9613
Accuracy	0.9768	0.9864	0.9857	0.9806	0.9907
IOU	0.0172	0.0724	0.2448	0.9347	0.9461
Recall	0.0174	0.0777	0.2657	0.9136	0.9702
Interfering Time (fps)	22.33	21.07	17.56	9.15	7.26
FLOPs (G)	-	-	-	60.24	63.44

* “√” indicates that the corresponding component is used, and “-” means that it is not used.

**Table 3:** Comparisons of different models.

Method	Precision	Accuracy	IOU	Recall	Training Time (s)	Interfering Time (fps)
Inter-frame difference	0.5939	0.9871	0.0954	0.1020	-	80.78
ViBe	0.5748	0.9827	0.2112	0.2503	-	7.91
KNN	0.5180	0.9875	0.0506	0.0531	-	21.07
Method in the literature [33]	0.0	0.9873	0.0	0.0	-	10.06
R2UNet	0.9985	0.9947	0.0184	0.0185	1913.43	6.46
Att UNet	0.9428	0.9895	0.9131	0.9626	858.91	7.47
Ours	0.9692	0.9987	0.9481	0.9775	916.15	7.26

**Table 4:** Results of the image quality evaluations.

Evaluation metrics	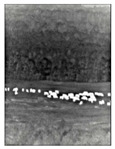 Img1	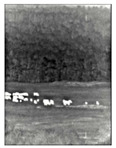 Img2	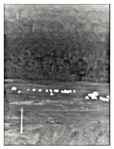 Img3	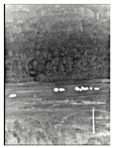 Img4
Brenner	80,664,844	82,031,387	112,356,710	118,895,951
Roberts	163,247,568	160,109,764	216,976,433	237,461,410
SMD2	16,377,601.0	17,695,173.0	25,543,868.0	27,485,529.0

**Table 5:** Results of evaluation metrics for different quality images.

Image	Precision	Accuracy	IOU	Recall
Img1	0.9415	0.9990	0.9101	0.9646
Img2	0.9236	0.9946	0.9046	0.9503
Img3	0.9089	0.9992	0.8896	0.9767
Img4	0.9322	0.9985	0.9052	0.9658

**Table 6:** Results of evaluation metrics for different scenes.

Picture	Precision	Accuracy	IOU	Recall
Img5	0.9357	0.9821	0.9014	0.9656
Img6	0.9012	0.9891	0.9159	0.9624
Img7	0.9123	0.9932	0.9026	0.9423
Img8	0.9115	0.9887	0.9244	0.9761

## Data Availability

The data presented in this study are available on request from the corresponding author, Haoting Liu.
